# Parent–child interaction therapy for preschool children with disruptive behaviour problems in the Netherlands

**DOI:** 10.1186/1753-2000-6-24

**Published:** 2012-06-13

**Authors:** Mariëlle E Abrahamse, Marianne Junger, E Lidewei Chavannes, Frederique J G Coelman, Frits Boer, Ramón J L Lindauer

**Affiliations:** 1 De Bascule, Academic Center for Child and Adolescent Psychiatry, Amsterdam, The Netherlands; 2 Department of Social Safety Studies, Institute for Innovation and Governance Studies (IGS), School of Management & Governance, University of Twente, Enschede, The Netherlands; 3 Department of Child and Adolescent Psychiatry, Academic Medical Center, University of Amsterdam, Amsterdam, The Netherlands

**Keywords:** Disruptive behaviour problems, Preschoolers, Parent–child interaction, Parent training, Psychotherapy

## Abstract

**Background:**

Persistent high levels of aggressive, oppositional and impulsive behaviours, in the early lives of children, are significant risk factors for adolescent and adult antisocial behaviour and criminal activity. If the disruptive behavioural problems of young children could be prevented or significantly reduced at an early age, the trajectory of these behavioural problems leading to adolescent delinquency and adult antisocial behaviour could be corrected. Parent–Child Interaction Therapy (PCIT) is a short-term, evidence-based, training intervention for parents dealing with preschool children, who exhibit behavioural problems. Recently, PCIT was implemented in a Dutch community mental health setting. This present study aims to examine the short-term effects of PCIT on reducing the frequency of disruptive behaviour in young children.

**Methods:**

This study is based on the data of 37 referred families. Whereby the results of which are derived from an analysis of parent reports of the Eyberg Child Behavior Inventory (ECBI), obtained during each therapeutic session. Furthermore, demographic information, extracted from client files, was also utilized. However, it must be noted that eleven families (27.5%) dropped out of treatment before the treatment protocol was completed. To investigate the development of disruptive behaviour, a non-clinical comparison group was recruited from primary schools (*N* = 59).

**Results:**

The results of this study indicate that PCIT significantly reduces disruptive behaviour in children. Large effect sizes were found for both fathers and mothers reported problems (*d* = 1.88, *d* = 1.99, respectively), which is similar to American outcome studies. At post treatment, no differences were found concerning the frequency of behavioural problems of children who completed treatment and those who participated in the non-clinical comparison group.

**Conclusion:**

The findings of this study suggest that PCIT is potentially an effective intervention strategy for young children and their parents in the Dutch population. However, further research into the evaluation of PCIT using a randomised controlled trial is recommendable.

## Background

Child disruptive behaviour disorders (DBDs), namely, conduct disorder (CD), and oppositional defiant disorder (ODD) as described by DSM-IV [1], are among the most common reasons for referring children and adolescents to mental health services [2]. Often, DBDs co-occur with attention deficit hyperactivity disorder (ADHD) [3]. Children with persistent high levels of aggressive, oppositional, and impulsive behaviours early in life are at a higher risk of serious adolescent and adult antisocial behaviour and criminal activity [4,5]. Although the prevalence rates of DBDs in the Dutch population has only been studied to a certain degree, one study concerning the prevalence of child psychiatric diagnoses of children between the ages of 6 and 8, using a structured diagnostic interview, revealed a mean prevalence rate of 12.8% for DBDs; 9.3% for girls and 15.2% for boys [6].

Within the last twenty years, several predictors and origins of DBDs have been identified. Most often, disruptive behaviour problems start in early childhood. Important risk factors relating to the development of chronic child disruptive behaviour problems can manifest during pregnancy and are often related to the history of a mother’s social adjustment and lifestyle during pregnancy [7]. Moreover, the transition from preschool to elementary school years is a critical period for the further development of aggressive behaviour, which may persist over time if not treated [8-10]. The development of DBDs in young children and their consistency can be explained by an interplay of genetic and environmental risk factors [11]. Given the early development of disruptive behaviour problems and their stability, as well as long term negative outcomes, prevention and intervention at an early stage is important and more likely to be (cost)effective [12,13]. It can be expected that interventions which target young children who are at a high risk of chronic disruptive behaviour problems at an early age, will have a more significant impact, compared to interventions which are carried out five to ten years later, when behavioural problems may have become persistent [9,13].

If disruptive behaviour problems of young children could be prevented or significantly reduced early in life, the trajectory of early disruptive behaviour problems leading to adolescent delinquency and adult antisocial behaviour could also be prevented. Unfortunately, therapeutic approaches targeting children with disruptive behaviours struggle with two main issues. First, the majority of them lack empirical evidence [14], and second, most target older children, such as pre-adolescents or adolescents, thereby missing a crucial age group in which prevention and intervention is of utmost importance [7,13]. Currently, parent training programs, which use parents as the primary agent of change, are the most effective method in reducing disruptive behaviours in young children [15]. A review of the effects of early parent training programs aimed at preventing antisocial behaviour and delinquency, shows that parent training is an effective intervention strategy in reducing child disruptive behaviour, with a mean effect size of 0.35. However, this effect size still indicates a small to moderate effect [16]. Although parent training programs are an effective treatment for children with behavioural problems, further research is required [17].

### Parent–child interaction therapy

Parent–Child Interaction Therapy (PCIT) [18] is a short-term, evidence-based parent training intervention which is used widely as a treatment for young children with disruptive behaviour problems. This treatment is based upon social learning [19], as well as attachment theory [20] and its primary aim is to change dysfunctional parent–child interactions into those that can be characterized as authoritative parenting [21,22]. The treatment is designed to help parents build a warm and responsive relationship with their child and to manage their child’s behaviour more effectively [23].

Several studies, mainly in the United States, have provided empirical evidence which indicated the effectiveness of PCIT, namely the improvement of parenting skills and the way parents interact with their children, as well as parental well-being, and the reduction of child disruptive behaviour with medium to large effect sizes [24]. Thereby, a body of evidence is growing on the effectiveness of PCIT to prevent child maltreatment [25]. PCIT has also proven to be robust across various groups and diagnoses. For instance, PCIT has been successfully adapted to meet the needs of several different cultural and language groups, including Puerto Rican [26], Mexican American [27], and Chinese [28]. Beside the cross cultural implementation of PCIT, PCIT has also explored new research directions including studies which work with several adaptations of the treatment which can in turn be used for different target groups. For example, PCIT has been tailored for physically abusive parents [29], prematurely born children [30], children with separation anxiety [31], and children with mental retardation [32].

In the past decade, the implementation of PCIT has expanded to several countries. However, evidence which illustrates the effectiveness of PCIT among children from other cultural backgrounds remains limited [33]. Although PCIT has been implemented in a number of European countries (e.g. the United Kingdom, Germany, Norway and Russia) [34], no evaluation studies are available in Europe. In the Netherlands, PCIT has been implemented in a community mental health setting in child and adolescent psychiatry since 2007. Most treatment outcome studies have been conducted at university clinics. Currently, the transferability of PCIT to community and other clinical settings is an important issue in evidence-based clinical practice. Delivering treatment in community mental health settings is often more challenging, and high rates of premature dropouts can limit its effectiveness. More research on PCIT is needed to examine the effectiveness of PCIT in real world clinics [35,36].

### Aim of the study

The present study describes the results of a preliminary evaluation of the short-term effectiveness of Parent–Child Interaction Therapy in the Netherlands which aims to reduce the disruptive behaviour of children. In a retrospective design, child disruptive behaviour was measured with the Eyberg Child Behavior Inventory (ECBI) [37]. We hypothesized that PCIT will have positive effects on the disruptive behaviour of young children.

## Methods

### Participants

Since the implementation of PCIT in a Dutch mental health setting, between January 2007 and July 2009, forty families were referred on the grounds of child disruptive behaviour. All of the families were contacted to provide permission for using their reports of the Eyberg Child Behavior Inventory (ECBI) [37] in this study. Because three families did not give their consent, data from 37 families were used in statistical analyses. Although the families who did not give their consent were composed of two-parent families, no significant differences were found in regard to other important demographic characteristics and scores on the ECBI at pre and post assessment between these three families and the participating families.

A total of 37 families formed the clinical group (Table 1). All of the participating families lived in or nearby Amsterdam, The Netherlands. In addition, as determined by a child psychiatrist, 17 children (45.9%) met the diagnostic criteria according to the fourth edition of the Diagnostic and Statistical Manual of Mental Disorders (DSM-IV) [1]. Only four children met the criteria for ODD only, six children for ADHD and only two children met the criteria for ASD (Autism Spectrum Disorder). Five children had co-morbid diagnoses. Two children met the criteria for both ADHD and ODD, two children met the criteria for ADHD, ODD and ASD, and one child met the criteria for ADHD and ASD. In all cases, a female caregiver/mother was involved in the treatment. In regards to fathers, 19 (51.4%) were involved in treatment sessions. Twenty-one children (56.8%) lived in two-parent families with their biological parents, and two children (5.4%) in this group were co-parented, meaning that the child lived with either divorced or separated parents, but in different homes. Thirteen children (35.1%) lived in single-mother families and three children (8.1%) had foster parents. The racial/ethnic composition of mothers was as followed; 62% Caucasian, 11% Surinamese, 8% Moroccan, 3% Turkish, and 16% from other, mainly African, countries.

**Table 1:** Desriptive statistics of the Treatment and Non-Clinical Comparison Groups

	Mean (*SD*) or Percent	
	TT (*n* = 37)	NC (*n* = 58)
Child age (years)	4.7 (1.5)	5.2 (0.8)
Child sex (% male)	75.5	50.8
Mother age (years)	34.9 (6.7)	36.3 (4.1)
Family status (% single parent)	35.1	1.7
Mother racial composition (% Caucasian)	62.0	96.6

*Note. TT* Total Treatment Group, *NC* Non-Clinical Comparison Group.

In order to investigate the development of disruptive behaviour over a period of six months, a non-clinical comparison group was recruited which consisted of children from the same age category as those from the clinical group. These families were recruited by students on primary schools. The mothers in this group filled out the ECBI twice over a six month period (*N* = 59), and this group was composed of 30 boys and 29 girls (Table 1). No significant differences (*p* < .05) were found between the ages of the mothers and children in the non-clinical group and the clinical group. Although there was a significant difference in gender composition between the clinical and non-clinical group, there were no gender differences on the mean ECBI scores on all presented scales.

### Measures

#### Eyberg child behavior inventory (ECBI)

The ECBI [37] is a 36-item parent report, which measures the degree of behavioural problems of children between the ages of 2 to 16. The ECBI assesses the behaviour on two different scales, the Intensity scale and the Problem scale. The ECBI Intensity scale measures the frequency of disruptive behaviour along a 7-point scale (1 = *never* to 7 = *always*), and the ECBI Problem scale measures whether or not parents view those behaviours as problematic (1 = *yes*, 0 = *no*). Several studies have demonstrated that both scales of the ECBI demonstrate a high level of reliability and validity in terms of measuring the disruptive behaviour of children [38,39]. Our study used a Dutch version translated by Raaijmakers, Posthumus, and Matthys (University of Utrecht, The Netherlands)*.* The norms for a clinical range were used from the professional manual [37]. Scores above 132 on the intensity scale and above 15 on the problem scale were considered clinically significant. Both parents completed the ECBI if the father was involved in the treatment sessions. Therefore, for the pre and post assessment data, ECBI reports of the first session (orientation) and last treatment session (graduation) were used.

### Procedure

All participating families received PCIT delivered in the Dutch language by one of the eight therapists who were trained in two workshops by the program developers. They attended the first workshop at the University of Florida and the second at the University of Oklahoma. The original treatment manual [40] was translated into Dutch. Each therapist had a Bachelor’s or Master’s degree in mental health related fields and had experience in clinical work. Therapists started their cases right after the training workshop. Throughout the training and during follow-up consultations, a strong emphasis was put on treatment fidelity. For supervision purposes, all therapy sessions were videotaped. Although treatment adherence was not formally assessed, additional supervision sessions were provided. Due to the fast implementation process and organizational limitations, this study was retrospective. After the termination of PCIT, all parents were asked for their permission to use their reports of the ECBI [37] conducted during treatment, and some demographic information from the client-files for scientific research.

### Treatment

Parent–Child Interaction Therapy (PCIT) is an intervention which focuses on children with disruptive behaviour problems and their caregivers [41]. PCIT consists of two phases of treatment, Child-Directed Interaction (CDI) and Parent-Directed Interaction (PDI). The first phase focuses on enhancing the parent–child relationship and the second on improving child compliance. Both treatment phases begin with a didactic parental teaching session followed by weekly sessions whereby the parent is coached by the therapist during play sessions with their child. The therapist provides the parent with feedback on their skills from an observation room behind a one-way mirror, via a bug-in-the-ear. Parents practice specific communication skills and behaviour management with their children. PCIT is customized per case and although it is often a short-term intervention, PCIT is not time-limited. In each session parent–child interactions are coded at the beginning to determine the family’s progress toward pre-established mastery criteria. Parents have to master the CDI criteria before starting with the PDI phase of treatment. The PDI phase continues until parents reach the mastery criteria for the PDI skills and rate their child’s behaviour well within a normal range. A consequence of this approach is that the number of sessions may vary among families. Nevertheless, each family receives the number of sessions necessary to master CDI and PDI skills in order to demote their child’s behaviour below clinical levels [34].

### Statistical analysis

The effectiveness analyses were performed on a sample of participants who completed the treatment. Paired samples *t*-tests were conducted on the mean scores of both parent’s ECBI from pre and post assessments. If a score of a parent on the ECBI was missing on a pre or post assessment, the information of that parent was removed from the analyses for the particular scale. Effect sizes (Cohen’s *d*) were calculated by dividing the pre and post test mean by the pooled standard deviation, whereby 0.2 indicated a small effect, 0.5 a medium effect, and 0.8 and higher a large effect size [42]. In all of the analyses, a two-tailed test was used and all *p* values < .05 were considered to be statistically significant. To determine whether the changes in disruptive behaviour in children were clinically significant, reliable change indices (RCI) [43] for each child were calculated by dividing the magnitude of change on the ECBI scales between pre and post assessment by the standard error of the difference score. Published norms for the ECBI clinical cut-off were used [38].

## Results

### Descriptive statistics

Out of the 40 participating families who started with PCIT, 11 families (27.5%) dropped out before treatment was completed, and seven families (63.6%) dropped out within the first ten sessions of treatment. There were several reasons that caused families to terminate treatment prematurely. Four families required other, more intensive treatment (36.4%), and two families (18.2%) disagreed with the treatment approach, particularly the time-out procedure in the Parent-Directed Interaction phase. Another two families (18.2%) simply stopped showing up for treatment, another family (9.1%) was too busy to participate, one family (9.1%) had to stop treatment due to severe parental relational problems and for one family (9.1%), the child’s behaviour improved enough to terminate treatment before meeting all skill levels by the parents.

Those families who did complete treatment (*n* = 26), went through a number of treatment sessions ranging from 10 to 38 sessions per family (*M* = 17.4, *SD* = 6.9). Most families (80.8%) finished PCIT within 10 to 20 treatment sessions. The mean duration of the Child-Directed Interaction phase was 10 sessions (*SD* = 5.2) and for the Parent-Directed Interaction phase the mean duration was 7 sessions (*SD* = 2.6). The mean duration of PCIT measured in time was 6.6 months (*SD* = 2.7), ranging from 3 to 12 months, per family.

### Outcomes of disruptive behaviour

Paired samples *t-*tests of pre and post measures revealed a significant reduction of the frequency of disruptive behaviour in children after treatment completion. Table 2 illustrates that at the end of the Child-Directed Interaction phase a significant decrease on both ECBI scales was already visible for both mothers and fathers. Overall, effect sizes between 1.48 and 1.99 at post-assessment were found for PCIT on child behavioural problems.

**Table 2:** Changes on the Eyberg Child Behavior Inventory (ECBI)

	*n*	Pre		Post		*t*	Effect size	*n*	Pre		Post		*t*	Effect size
		*Intensity*		*Intensity*			*(d)*		*Problem*		*Problem*			*(d)*
		*M*	*SD*	*M*	*SD*				*M*	*SD*	*M*	*SD*		
**Mothers**														
CDI	25	156.4	32.0	128.2	28.9	6.2^***^	.92	23	20.4	8.3	17.3	8.0	2.5^**^	.38
PDI	24	127.3	28.3	102.8	23.7	4.5^***^	.94	22	16.5	7.5	7.9	6.7	4.8^***^	1.21
Total treatment	23	154.0	32.2	100.2	20.5	8.4^***^	1.99	21	20.0	8.5	7.8	6.9	5.6^***^	1.56
**Fathers**														
CDI	14	151.9	31.8	128.9	34.8	2.6^**^	.69	12	21.4	6.8	15.8	10.1	2.2^*^	.65
PDI	16	126.4	31.9	101.9	31.2	3.3^*^	.78	15	15.5	9.3	7.9	8.9	3.8^**^	.83
Total treatment	15	153.3	30.9	101.0	24.3	6.7^***^	1.88	12	19.8	7.2	8.0	8.9	5.9^***^	1.48
**Non-clinical group**^**1**^	59	80.5	20.4	80.8	22.8	-.2	-.02	56	3.3	5.3	2.3	4.2	1.8	.21

*Note. ECBI* Eyberg Child Behavior Inventory, *CDI* Child-Directed Interaction phase, *PDI* Parent-Directed Interaction phase.

^*^p < .10, ^**^p < .05, ^***^p < .001.

^1^ Post in the Non-Clinical Comparison Group corresponds to a six months follow-up; This Non-Clinical Group only represents mothers.

In the non-clinical comparison group, no behavioural changes were reported at the six-month follow-up assessment. When the clinical group mothers were compared with the non-clinical group mothers on the ECBI Intensity scale at post treatment, no significant differences were found between the groups. However, mothers in the clinical group continued to view their child’s behaviour as significantly more problematic (ECBI Problem Scale; *t* (81) = 2.21, *p <* .05) than mothers in the non-clinical comparison group.

**Figure 1:**
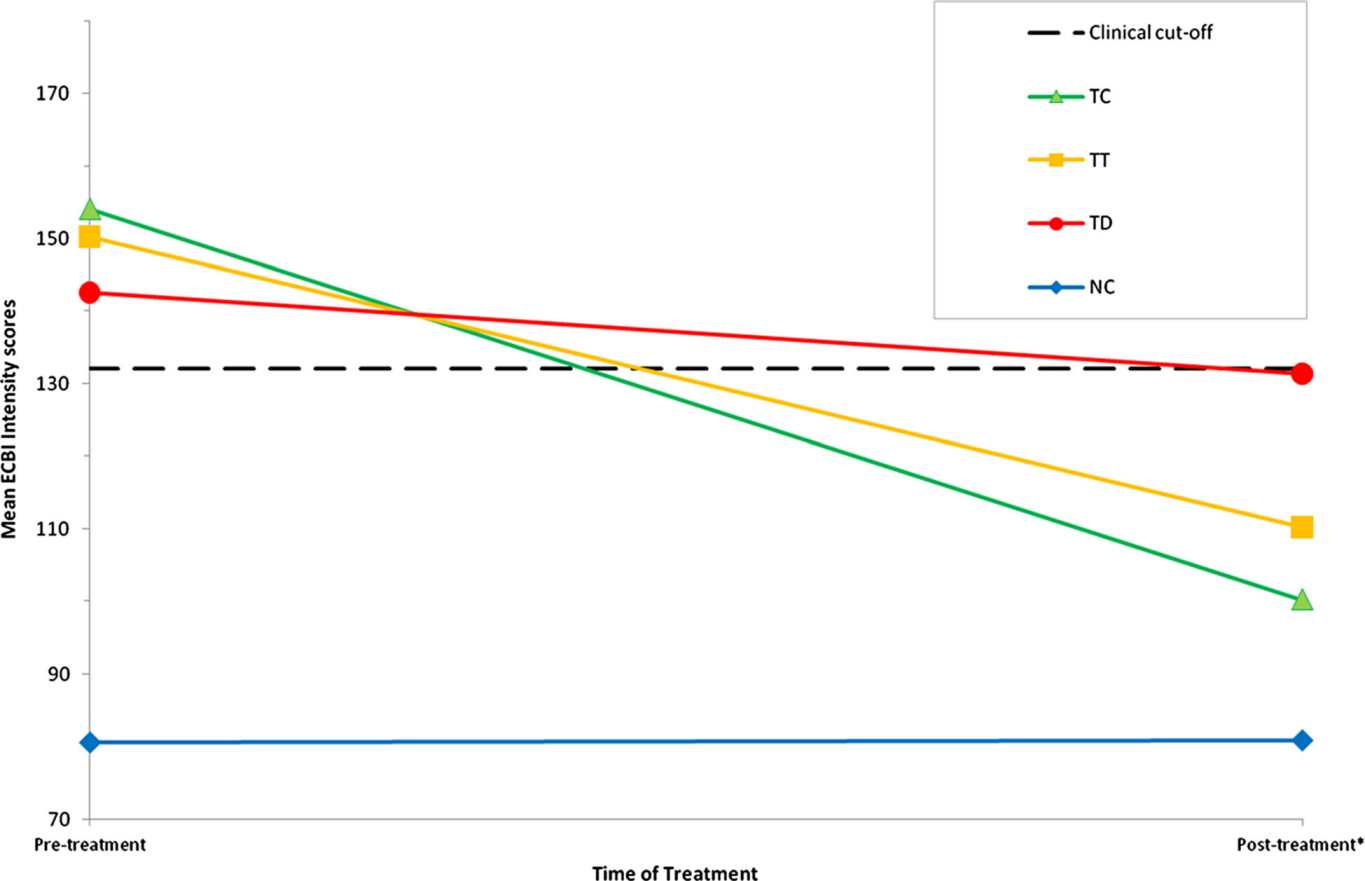
**Mean scores on the Intensity scale on the Eyberg Child Behavior Inventory (ECBI) for mothers in groups.** *Post = Post-treatment or six months follow up for the non-clinical comparison group. TC = Treatment Completers Group (*n* = 23); TT = Total Treatment Group (dropouts included) (*n* = 34); TD = Treatment Dropout Group (*n* = 11); NC = Non-Clinical Comparison Group (*n* = 59)

Figure 1 illustrates the mean scores of the ECBI Intensity scales for mothers in the different groups. This figure also includes the means of the total treatment group including the dropouts (*n* = 34) and the families who dropped out of treatment (*n* = 11) separately. Even when the dropouts are included, the means on the ECBI Intensity scale significantly improved from pre treatment to post treatment (Total Treatment Group; *t* (33) = 6.81, *p <* .001), and large effect sizes where obtained (*d =*1.15). Although Figure 1 shows a decrease in means between pre and post assessment for the families who dropped out of treatment prematurely, no significant differences were found in this group.

### Clinical significance

In order to measure individual change, the reliable change index [43] was calculated (Table 3). Participants of both the completer and dropout groups were classified according to the criteria of Jacobson et al. [44], and were presented in the same way as in Thomas and Zimmer-Gembeck [25]. In addition, based on the U.S. norms of the ECBI presented in the professional manual [37], 81.4% of the mothers of the total treatment group rated their child’s behaviour at pre assessment in the clinical range on one or both of the ECBI scales. After terminating PCIT, 29.7% of the mothers of this total group (dropouts included) still rated their child’s behaviour within the clinical range.

**Table 3:** Frequencies and percentages of Treatment Completers and Dropouts in Reliable Change Index (RCI) Categories

	**Recovered**		**Improved**		**Unchanged**		**Deteriorated**		**False Positive**	
	*Completer*	*Dropout*	*Completer*	*Dropout*	*Completer*	*Dropout*	*Completer*	*Dropout*	*Completer*	*Dropout*
**Mothers**										
ECBI Intensity	17 (73.9)	2 (18.2)	0 (0.0)	0 (0.0)	4 (17.4)	7 (63.6)	0 (0.0)	1 (9.1)	2 (8.7)	1 (9.1)
ECBI Problem	15 (71.4)	2 (25.0)	0 (0.0)	0 (0.0)	6 (28.6)	5 (62.5)	0 (0.0)	0 (0.0)	0 (0.0)	1 (12.5)
ECBI Intensity	10 (71.4)	-	1 (7.1)	-	3 (21.4)	-	0 (0.0)	-	0 (0.0)	-
ECBI Problem	8 (66.7)	-	1 (8.3)	-	3 (25.0)	-	0 (0.0)	-	0 (0.0)	-

*Note. ECBI* Eyberg Child Behavior Inventory; Scores > 132 on the Intensity scale and > 15 on the Problem scale were considered as clinically significant

*Recovered* Passed RCI and clinical significance; *Improved* Passed RCI but no clinical significance, *Unchanged* Unchanged RCI and unchanged or deteriorated clinical significance, *Deteriorated* Deteriorated in both RCI and clinical significance, *False Positive* improved clinical significance but unchanged RCI; RCI > 1.96 = Reliable Change Index improved and recovered categories.

^1^The only father in the dropout group had missing values on the pre-assessment.

Using the reliable change index, most mothers (73.9%) reported a change in the frequency of their child’s disruptive behaviour, whereby their child’s behaviour was rated within the range of normal functioning. Nevertheless, 17.4% of the mothers who completed treatment still did not report a reliable change in their child’s behaviour.

Although eleven families dropped out of treatment before completing treatment protocol, two families (18.2%) in this group were still classified as recovered. However, most families who dropped out of treatment reported insufficient or even a negative change in their child’s behaviour.

## Discussion

Our study supports our hypothesis that Parent–Child Interaction Therapy (PCIT) has positive effects on the disruptive behaviour of Dutch preschoolers. The study indicates that behavioural problems declined significantly during treatment. After the implementation, 40 families were treated with PCIT and 37 of those were included in this present study. The majority of families (72.5%) finished treatment protocol, however 27.5% dropped out after having participated in at least one session.

After treatment completion, most of the parents reported a significant reduction in the behaviour problems of their child. The effect sizes of the reduction of their child’s disruptive behaviour problems were large, varying between 1.48 and 1.99 and were comparable with the effect sizes as reported in a meta-analysis on PCIT where they varied between 1.21 and 1.57 on the two ECBI scales [24]. Therefore, at post treatment almost all parents reported their child’s behaviour in the range of normal functioning, and which did not differ from the non-clinical comparison group.

In regards to the ECBI Intensity scale mean ratings of the non-clinical group, it is worth mentioning that these means indicate that Dutch ECBI norms differ from those mentioned in U.S. samples. However, these current findings are similar to other European ECBI standardization studies, which also found lower means on the ECBI [45,46]. Although it would be recommendable to study the Dutch ECBI norms in a larger sample, the differences between norms, as compared to the U.S. samples, may also lead to a reconsideration of the ECBI norms of normal functioning in the Dutch PCIT manual.

In over 50% of the total cases, father involvement was achieved. Father reports of child disruptive behaviours at pre and post treatment were similar to those of the mothers. Even though father ratings are not often reported in treatment outcome studies [47], this finding suggests that fathers could profit from their involvement in treatment the same way that mothers do. The present findings are similar to the results of Schuhmann et al. [23] who also included fathers and analysed these results separately.

The results of individual changes show that even for families who dropped out before treatment protocol was completed, PCIT can be a sufficient intervention strategy for reducing child behavioural problems. However, the results also conveyed that after completing PCIT, a small group of parents still reported the behaviour of their child to be within the clinical range. These results indicate that although some parents had reached the mastery skills of the PDI phase, PCIT was terminated before their child’s behaviour was ranked within the normal range of functioning, which was also part of the PCIT termination procedure. This suggests that therapists need to obtain additional training in order to follow up on the PCIT protocol accurately. In this current study adherence to the treatment manual was not formally assessed. Future research should address this issue.

### Strengths and limitations

Our study examined the service delivery of an evidence-based treatment in a mental health community setting. This contributes to bridging the gap between research-based approaches and routine practice. It thereby also contributes to the literature on evidence-based treatments for children with disruptive behaviour problems. Given the diversity of the sample, whereby 38% was categorized as non-western, this current study also contributes to the knowledge of the effectiveness of PCIT for immigrant families and families of non-western origin. It would be recommendable to study this specific group more extensively in further research.

However, there are also a number of limitations inherent to this study. Although the non-clinical comparison group provided valuable information about the stability and the frequency of behaviour problems in this non-clinical group, no clinical control group was available and long-term effectiveness of treatment was not measured. Due to the absence of a clinical control group, improvements due to maturational or other factors could not be ruled out. However, disruptive behaviour problems of young children have a high degree of stability over time if not treated [8,9]. Regarding the large effect sizes on the decrease of reported child behaviour problems and the high stability of the behaviour of children in the non-clinical comparison group in this study, it seems unlikely that the improvements were simply spontaneous.

Second, due to the retrospective design of this current study only parent-reports (ECBI) were available for the measurement of treatment outcome effects. As mentioned earlier, the lack of Dutch norms for the ECBI have consequences for the interpretations of the results in the Dutch context. Thereby, the normal range of functioning of a child on the ECBI is a part of the mastery criteria to terminate PCIT. Hence, more information on parent personality characteristics, parenting stress and child behaviour would provide a wider range of information for the treatment outcomes. This information is highly recommended for future research to address questions concerning the effectiveness of PCIT on other parent and child functioning areas. Furthermore, observational measures using the Dyadic Parent–child Interaction Coding System (DPICS) [48] are recommended for providing more information about the behaviours, as well as the quality of parent–child interactions. The inclusion of a diagnostic interview for concerning child behavioural problems and the use of more independent sources (e.g. teachers) could have also improved the study.

The attrition rate (27.5%) in the current study was similar or slightly lower than other U.S. PCIT studies carried out in community mental health settings [35,49]. However, the attrition rate is still high and research is needed to identify the characteristics of specific families that are at risk of treatment drop out. Thus, more support from therapists and other professionals is needed to help high-risk families stay engaged and complete the treatment program. Nevertheless, the results do indicate that a premature termination of PCIT does not have to lead to negative outcomes on child behaviour in all cases.

The limitations of this study can be associated with the preliminary nature of the research and can also be identified as a consequence of a fast implementation process.

## Conclusions

Despite the limitations of this study, it does provide significant evidence of short-term effectiveness of PCIT in the Netherlands. Nonetheless, future research is required to address the shortcomings of the present study. A randomised controlled trial is recommended for a further evaluation of PCIT, which can compare the results with a clinical control group and assess long-term effectiveness. Furthermore, studies in community mental health settings are necessary for obtaining knowledge about treatment effectiveness in a challenging population. Determining effective strategies for reducing treatment attrition is also important in these settings. Given the limited knowledge at this time, our findings are a step forward in the evaluation of PCIT as a promising intervention strategy in reducing child disruptive behaviour problems in the Netherlands.

## Competing interests

The authors declare that they have no competing interests.

## Authors’ contributions

MA was involved in the data collection, performed the statistical analysis and drafted the manuscript. FC and LC participated in the design and data collection of the study. MJ and RL participated in the planning, supervision and co-ordination of the study as well as the critical revision of the draft of the manuscript. FB also critically revised the draft of the manuscript. All of the authors have read and given their approval to the final manuscript.
